# Comparison of loop-mediated isothermal amplification assay and smear microscopy with culture for the diagnostic accuracy of tuberculosis

**DOI:** 10.1186/s12879-016-2140-8

**Published:** 2017-01-17

**Authors:** Baye Gelaw, Yitayal Shiferaw, Marta Alemayehu, Abate Assefa Bashaw

**Affiliations:** 1 Department of Medical Microbiology, School of Biomedical and Laboratory sciences, CMHS, University of Gondar, P.O. box 196, Gondar, Ethiopia; 2 Department of Microbiology, Parasitology and Immunology, Faculty of Medicine, Addis Ababa University, Addis Ababa, Ethiopia; 3 School of Medicine, Flinders University, Bedford Park, SA 5042 Adelaide, Australia

**Keywords:** Loop-mediated isothermal amplification, Tuberculosis, Smear microscopy, Culture

## Abstract

**Background:**

Tuberculosis (TB) caused by *Mycobacterium tuberculosis* is one of the leading causes of death from infectious diseases worldwide. Sputum smear microscopy remains the most widely available pulmonary TB diagnostic tool particularly in resource limited settings. A highly sensitive diagnostic with minimal infrastructure, cost and training is required. Hence, we assessed the diagnostic performance of Loop-mediated isothermal amplification (LAMP) assay in detecting *M.tuberculosis* infection in sputum sample compared to LED fluorescent smear microscopy and culture.

**Method:**

A cross-sectional study was conducted at the University of Gondar Hospital from June 01, 2015 to August 30, 2015. Pulmonary TB diagnosis using sputum LED fluorescence smear microscopy, TB-LAMP assay and culture were done. A descriptive analysis was used to determine demographic characteristics of the study participants. Analysis of sensitivity and specificity for smear microscopy and TB-LAMP compared with culture as a reference test was performed. Cohen’s kappa was calculated as a measure of agreement between the tests.

**Results:**

A total of 78 pulmonary presumptive TB patients sputum sample were analyzed. The overall sensitivity and specificity of LAMP were 75 and 98%, respectively. Among smear negative sputum samples, 33.3% sensitivity and 100% specificity of LAMP were observed. Smear microscopy showed 78.6% sensitivity and 98% specificity. LAMP and smear in series had sensitivity of 67.8% and specificity of 100%. LAMP and smear in parallel had sensitivity of 85.7% and specificity of 96%. The agreement between LAMP and fluorescent smear microscopy tests was very good (κ = 0.83, *P*-value ≤0.0001).

**Conclusions:**

TB-LAMP showed similar specificity but a slightly lower sensitivity with LED fluorescence microscopy. The specificity of LAMP and smear microscopy in series was high. The sensitivity of LAMP was insufficient for smear negative sputum samples.

## Background

Tuberculosis (TB) caused by *Mycobacterium tuberculosis* remains one of the leading causes of morbidity and mortality, particularly in developing countries [1]. The human immunodeficiency virus (HIV) epidemic worsened the TB situation by accelerating the progression from primary infection to disease [2]. Diagnosis of TB involves clinical evaluation, including chest radiography and bacteriological tests. Bacteriological diagnosis depends on smear microscopic examination and bacterial cultures, which has been the traditional method for many decades [3]. Although sensitivity is insufficient, Ziehl-Neelsen (ZN) smear microscopy is widely used due to its rapidity and inexpensiveness [4]. The sensitivity of sputum ranges from 34–80% and is lowest in sputum with low bacilli load [5]. Because of the limitations of conventional light microscopy using ZN method, auramine O stained fluorescence microscopy that improves sensitivity and takes less time to perform has been introduced. However, fluorescence microscopy has reduced specificity and its feasibility issue yet didn’t replace ZN method [6–8]. In addition among HIV-coinfected individuals symptomatic screening, chest radiograph, and microscopic examination are less sensitive and specific [9].

In contrast, a sputum culture is considered the most accurate test due to its high sensitivity and specificity. However, this technique is labour-intensive and time-consuming. Nucleic acid amplification technique (NAAT) allows the rapid, sensitive and specific diagnosis of TB [10, 11]. The commonly used NAAT polymerase chain reaction (PCR) that includes extraction and amplification of target DNA are extremely prone to cross-contamination [7, 12]. Gene Xpert MTB/RIF NAAT has been recently developed for the rapid initial diagnosis of TB. This assay directly detects TB and rifampicin resistant *M. tuberculosis* in unprocessed samples [13]. However, the utility of current NAAT methods is limited by their cost, particularly in countries where TB is endemic [7, 13].

A test that combines the rapidity of microscopy and sensitivity of bacterial culture methods would facilitate the initiation of anti-TB treatment. Loop-mediated isothermal amplification (LAMP) that requires less sophisticated settings has recently been developed [14]. LAMP is a new TB detection method developed by Eiken Chemical Co., Ltd. in a joint development agreement with the Foundation for Innovative New Diagnostics, for use at the microscopy centre level to provide a rapid TB case detection [15]. LAMP amplifies DNA in less than an hour with high efficiency under isothermal conditions using six sets of primers [16]. The large amount of DNA generated and the high specificity of the reaction make it possible to detect amplification by visual inspection of fluorescence or turbidity, without the need for the usual DNA extraction, wash steps, gel electrophoresis or instrument detection of the labelled probe [15]. The use of a closed-tube system minimizes the risk of workspace contamination with amplicon [17].

TB-LAMP appears to be suitable and good candidate for use in developing countries for its sensitivity and rapidity [7]. Several LAMP assays targeting different *M.tuberclosis* genes have been evaluated for the diagnosis of TB and showed great improvement of both sensitivity and specificity. Suitably if confirmed, LAMP can potentially improve the diagnosis of TB in resource limited settings [18, 19]. It is recommended that further research is needed to assess LAMP assay especially in high-burden TB and HIV settings [15]. Hence, we assessed TB diagnostic performance of LAMP for the detection of *M. tuberculosis* from sputum samples compared with fluorescent smear microscopy and culture.

## Methods

### Study setting and population

A cross-sectional institution based study was conducted at the University of Gondar Hospital (UoGH) from June 01, 2015 to August 30, 2015. The hospital is found in Gondar town. Gondar town is located in the Semien Gondar zone of the Amhara Region, North of Lake Tana, Northwest Ethiopia, and 748 km far from Addis Ababa. The UoGH is a referral hospital with more than 400 beds serving a population of about 5 million people in Northwest Ethiopia. All presumptive TB patients attending UoGH were eligible for the study. Presumptive TB cases were identified through clinical and radiological routine TB examination in accordance with world health organization (WHO) national guidelines. Briefly, patients with productive cough for two or more weeks and accompanied by one or more symptoms of weight loss, loss of appetite, night sweats, chest pain, fever, shortness of breath, and fatigue and malaise are considered to be presumptive TB cases. Study subjects were recruited conveniently from consecutive TB suspected presumptive pulmonary TB patients attending the TB clinic during the study period.

### Data collection

After taking written informed consent, a questionnaire based interview of TB patients was used to collect demographic information. Presumptive TB patients were consented so as to bring three consecutive (spot-morning-spot) sputum samples for smear microscopy, TB culture and LAMP assay. Sputum samples were collected using dry, clean, leak proof and screwed plastic containers. Sputum samples were smeared directly on a slide and subjected to auramine O staining and LED fluorescent microscopy and examined by experienced laboratory technologists. The collected sputum samples were pooled and stored at −20 °C for LAMP and culture. Unprocessed pooled sputum samples stored at −20 °C were transported within a week to Bahir Dar Regional Health Research Laboratory Center using ice box for *M. tuberculosis* culture.

### TB-LAMP assay

The assay was performed based on the WHO reported LAMP procedure [15] by trained data collector. Using a wide-bore disposable pipette 60 μl of sputum was collected from a sputum cup and transferred to a heating tube containing extraction solution. After mixing by inverting 3–4 times, the heating tube was placed in heating block at 90 °C for 5 min to lyse and inactivate Mycobacteria. The heating tube was removed from the heating block and let it cool down for 2 min. The heating tube was attached to an adsorbent tube and mixed by shaking until all the powder has completely mixed with the solution. An injection cap was placed on the adsorbent tube and was screwed tightly to pierce the seal. The nozzle of injection cap was inserted into a reaction tube and drops of solution (30 μl) were transferred to the reaction tube. After confirming the temperature on the digital display on the incubator to be 67 °C, the reaction tubes was loaded into the heating block and started the reaction. The amplification was stopped automatically after 40 min. The reaction tubes were transferred into the fluorescence detector and record the results. Finally without opening the tubes reaction tubes were discarded by incineration.

### Sputum culture

Equal volume of 4% NaOH was added to the sputum sample and vortex mixed in a class I BSC to decontaminate the sputum. One ml of the decontaminated sputum sample was transferred into a centrifuge tube using sterile 1 ml pasture pipette and incubated at room temperature for 10 min on the shaker to free the bacilli. This was centrifuged at 3000 revolution per minute for 15 min. The supernatant was decanted, the sediment mixed and pellet was re-suspended in 1–2 ml of phosphate buffer saline. One drop of phenol red indicator was added followed by neutralizing the sediment by adding 2 N HCl drop by drop with continuous shaking until the color changes from red to yellow. A 0.5 ml of neutralized sputa were inoculated to Lowenstein-Jensen (LJ) media using sterile disposable Pasteur pipette and incubated at 37°c in slant position for 1 week. After one week, the inoculated culture media were kept in an upright position and monitored weekly for growth for 8 weeks. Those culture media with sign of growth further confirmed by ZN smear microscopy and biochemical tests for *M. tuberculosis*.

### Data analysis

The data were initially recorded in a laboratory log book, then entered into a computer and checked for completeness and were analyzed using SPSS software - version 20. A descriptive analysis was used to determine demographic characteristics of the study participants. Sensitivity, specificity as well as positive and negative predictive values (PPV & NPV) were calculated for the LAMP and LED fluorescent smear microscopy in comparison with culture as the gold standard. LAMP and smear microscopy testing in series and parallel were compared against the culture. For this purpose, LAMP and smear microscopy were compared in series (LAMP performed only if smear positive and considered TB positive if both tests are positive or TB negative if either test is negative) and in parallel (LAMP performed for all samples and considered TB positive if either smear or LAMP is positive and TB negative if both are negative). Cohen’s kappa was calculated as a measure of agreement between the tests. Normally, κ value ranges from 0 to 1 and 1 implies perfect agreement whereas 0 is no agreement. We used the following interpretations: poor agreement <0.20; moderate agreement 0.40–0.60; good agreement 0.60–0.80; very good agreement ≥0.80.

### Quality assurance

All laboratory diagnostic procedures were carried out based on established routine laboratory diagnosis standard operation procedures by trained experienced laboratory staff working in the respective facilities. Patient samples were anonymized and only identified by their unique hospital and laboratory numbers. All data collectors involved in the laboratory investigations were blinded to the results of clinical examination and all other prior laboratory assays findings.

## Results

A total of 80 pulmonary presumptive TB patients were recruited in the current study. Two patients were excluded because of culture contamination. All the sputum samples were collected from subjects before anti-tuberculosis treatment initiation. Of these 78 TB suspects, 40(51.3%) were female. The mean age of the study subject was 37.3 years (±15.1SD). Majority of the study subjects 44(56.4%) were married. Forty seven (60.3%) of the study subjects had no formal education and 45(57.7%) of the study participants were rural dwellers. Among TB suspected patients the most common clinical manifestations were night sweats in 79 (94.9%), pulmonary infiltration in 53 (67.9%) and weight loss in 44 (56.4%) (Table 1).

**Table 1:** The demographic and clinical characteristics of TB suspected patients

Variable		Number	%
Sex	Male	38	48.7
	Female	40	51.3
	13–25	21	26.9
Age	26–35	22	28.2
	≥35	35	44.9
Residence	Urban	33	42.3
	Rural	45	57.7
	Single	26	33.3
Marital status	Married	44	56.4
	Divorced	8	10.3
	Illiterate	47	60.3
Educational status	Primary	13	16.7
	Secondary and above	18	20.1
Weight loss	Yes	44	56.4
	No	34	43.6
Night sweating	Yes	74	94.9
	No	4	5.1
Pulmonary infiltrate	Yes	53	67.9
	No	25	32.1
Cavity	Yes	13	16.7
	No	65	83.3

The performance of LAMP and smear LED fluorescent microscopy as compared to the LJ culture is presented in Table 2. The overall sensitivity and specificity of TB-LAMP for *M.tuberculosis* detection were 75 and 98% respectively for the entire samples when compared to culture as the reference standard. Whereas smear microscopy showed 78.6% sensitivity and 98% specificity as compared to culture method. However, LAMP showed a sensitivity and specificity of 82.6 and 94.5% respectively for smear positive specimens. The sensitivity and specificity of TB-LAMP for the diagnosis of TB from smear negative sputum samples were 33.3 and 100% respectively. On the other hand, for both culture and LAMP positive sputum specimens the sensitivity and specificity of smear microscopy were 90.4 and 93% respectively. Among both smear and culture positive sputa LAMP showed sensitivity of 86.4 and specificity of 94.6%. Of fifty culture negative sputum specimens, one was positive for LAMP and one for fluorescent smear microscopy.

**Table 2:** Comparison of LAMP, smear and culture methods for the diagnosis of pulmonary tuberculosis

	Culture+	Culture–	Se %(95% CI)	Sp %(95% CI)	PPV (%)	NPV (%)	κ
LAMP+	21	1	75(56.6–87.3)	98(89.5–99.6)	95.5	87.5	0.77
LAMP-	7	49					
Smear+	22	1	78.6(60.5–89.8)	98(89.5–99.6)	95.6	89.1	0.797
Smear-	6	49					
	Smear +	Smear -					
LAMP+	19	3	82.6(62.9–93)	94.5(85.2–98.1)	86.4	92.9	0.83
LAMP-	4	52					
	Culture + Smear +	Culture- Smear -					
LAMP+	19	3	86.4(66.7–95.3)	94.6(85.4–98.2)	86.4	94.6	0.82
LAMP-	3	53					
	Culture + Smear-	Culture-Smear+					
LAMP+	2	0	33.3(9.7–70)	100(20.7–100)	100	80	NA
LAMP -	4	1					
	LAMP+ Culture+	LAMP- Culture-					
Smear +	19	4	90.4(71.1–97.4)	93(83.3–97.2)	82.6	96.4	0.82
Smear-	2	53					

*Se* Sensitivity, *Sp* Specificity, *PPV* Positive predictive value. *NPV* Negative predictive value, *κ* Kappa value. *CI* confidence interval

The agreement between the tests was measured by Cohen’s kappa (κ) statistics. LAMP and smear microscopy methods showed a very good concordant result (κ = 0.83, *P*-value ≤0.0001). Good agreement between LAMP and culture result were also observed (κ = 0.77, *P* ≤0.0001) (Table 2). LAMP and smear microscopy testing in series and parallel were compared against TB culture as a reference standard. When both the smear microscopy and LAMP were performed serially, the overall sensitivity and specificity were 67.8 and 100.0% respectively. When both the fluorescent smear microscopy and LAMP were performed in parallel, we observed an overall sensitivity and specificity of 85.7 and 96% respectively. The agreement between culture result and the result from LAMP and smear in series were very good (κ = 0.83, *P* ≤0.0001). Moreover, when the two tests done in parallel, they showed good concordant results ((κ = 0.79, *P* ≤0.0001) (Table 3).

**Table 3:** Comparison of LAMP and smear in series and parallel for the diagnosis of pulmonary tuberculosis

		Culture+	Culture–	Se % (95% CI)	Sp % (95% CI)	PPV (%)	NPV (%)	κ
2tests in serial	Pos	19	0	67.8(49.3–82.1)	100(92.3–100)	100	84.7	0.83
	Neg	9	50					
2tests in parallel	Pos	24	2	85.7(68.5–94.3)	96(86.5–98.9)	92.3	92.3	0.786
	Neg	4	48					

*Se* Sensitivity, *Sp* Specificity, *PPV* Positive predictive value. *NPV* Negative predictive value, *κ* Kappa value, *CI* confidence interval

## Discussion

The absence of affordable, rapid and accurate TB point-of-care diagnostic tests especially in resource limited settings are jeopardized the control of the TB diseases. In spite of its low sensitivity, the sputum ZN microscopy is the main stay diagnostic method available for the detection of TB at peripheral laboratories in developing countries [20]. The development of rapid NAAT based tests has paved the way for generation of highly sensitive point-of-care tests. However, the utility of current NAAT methods are limited by their cost and complexity, particularly in TB high burden developing countries. Hence, we compared LAMP assay and smear LED fluorescent microscopy with culture as a reference standard test. As compared to culture the reference standard method, we found an overall specificity of 98% for both LAMP and LED fluorescent microscopy. However, the sensitivity of TB-LAMP (75%) was found to be slightly lower than the sensitivity of LED fluorescent microscopy (78.6%).

In this study, the sensitivity and specificity of LAMP compared to culture were comparable to findings reported from India [21]. The specificity of our LAMP assay was higher as compared to the LAMP specificity (94%) reported from the study done in Gambia. However, the finding from Gambia indicated a very high sensitivity (99%) of LAMP assay [22]. Our LAMP assay sensitivity (33.3%) in smear negative sputum samples was lower compared to the findings reported from reference laboratories in South Africa, Vietnam, Peru and Brazil (56%) [15]. However, this low sensitivity of LAMP assay among smear negative specimens is concordant with the findings identified in India (33.3%) [21].

In our study, fluorescent smear microscopy showed 78.6% sensitivity and 98% specificity compared to culture as reference standard. This sensitivity of smear microscopy is relatively comparable to finding (79.5%) reported from the Indian study [21]. In contrary to our finding, relatively higher sensitivity (84.7%) and comparable specificity (98.9%) of smear microscopy were documented in the finding reported from South Africa [8]. These discrepancies in the diagnostic performance of LAMP assay among reports might be confounded by differences in TB laboratory protocols and practices. Unlike our study, some laboratories performed LAMP assay using decontaminated sputum samples [21].

In the present study smear microscopy and LAMP tests in series had an overall sensitivity of 67.8% and specificity of 100.0%. However, we observed better sensitivity (85.7%) of smear microscopy and LAMP tests were done in parallel. This high specificity of smear microscopy and LAMP tests in series was in line with the result found in very recent study done in India [21]. LAMP and smear microscopy had very good agreement for the detection of pulmonary TB. Two specimens positive for either smear or LAMP were negative for culture. The possible explanation for LAMP or smear positive but culture negative results could be due to the existence of dead bacilli. The current LAMP procedures are not as simple as smear microscopy. Although the feasibility of LAMP was not evaluated in this study, the need for cold chain transportation system and costly LAMP reagents may hamper the application of the test in peripheral laboratories. Perhaps further modification of the LAMP method to optimize its practicability could be also important.

## Conclusions

LAMP showed similar specificity but a slightly lower sensitivity with LED fluorescence microscopy. The specificity of LAMP and smear in series was high. Although LAMP appears to be appropriate for detection of smear positive TB, the sensitivity of LAMP was insufficient for smear negative sputum samples. Therefore, to apply LAMP as an alternative method for the diagnosis of TB, further evaluation of its diagnostic performance among smear negative samples with large-scale study should be considered.

## Abbreviations

HIV: Human immunodeficiency virus
LAMP: Loop mediated isothermal amplification
NAAT: Nucleic acid amplification technique
NPV: Negative predictive value
PCR: Polymerase chain reaction
PPV: Positive predictive value
TB: Tuberculosis
UoGH: University of gondar hospital
WHO: World health organization
ZN: Ziehl-Neelsen
